# Malnutrition among lactating women in sub-Saharan Africa: an analytic review of spatial distribution, burden and determinants

**DOI:** 10.3389/fpubh.2025.1564581

**Published:** 2025-06-03

**Authors:** Bekahegn Girma, Azizur Rahman

**Affiliations:** ^1^School of Nursing and Midwifery, Debre Berhan University, Debre Berhan, Ethiopia; ^2^School of Computing, Mathematics and Engineering, Charles Sturt University, Wagga Wagga, NSW, Australia

**Keywords:** sub-Saharan Africa, lactating mothers, spatial distribution analysis, underweight, overweight/obesity

## Abstract

Malnutrition, encompassing both underweight and obesity, poses a significant public health challenge for women worldwide, spanning across developed and developing nations. Sub-Saharan Africa (SSA) bears a notably high burden of underweight, despite recent years have seen a noticeable increase in obesity rates. Lactating women are especially vulnerable to malnutrition. This analytic review aimed to compile current knowledge on the spatial distribution, prevalence, and contributing factors of malnutrition among lactating mothers in Sub-Saharan Africa, highlighting research gaps. This review systematically searched previous primary studies and reports from databases including PubMed, MEDLINE, PsycINFO, Web of Science, HINARI, EMBASE, African Journal of Online (AJOL), Scopus and Google Scholar. Various significant findings were synthesized in textual descriptions, figures and tables. The prevalence of underweight among lactating women in sub-Saharan Africa exhibits considerable disparity, spanning from 5.6 to 54.8%. However, there was no comprehensive summarized evidence for this issue in the region. Additionally, our findings emphasize a deficiency in comprehending the geographic distribution and factors influencing malnutrition among lactating women in sub-Saharan Africa. The burden of overweight in SSA was 15.9% among reproductive women; this burden is going to be increased. However, there were no studies conducted among the most vulnerable women, lactating women. Similarly, despite the spatial distribution of obesity/overweight among reproductive omen in SSA was known, there were no evidences for lactating women who have high risk for malnutrition as pregnant women. Hence, conducting population-wide, representative, and comprehensive research utilizing Demographic Health Survey data from countries in sub-Saharan Africa is imperative to fully comprehend the extent of the problem and effectively address the burden of malnutrition in this vulnerable population. Additionally, identifying hotspot areas of malnutrition specifically among lactating women within sub-Saharan Africa through spatial distribution analysis is essential for allocating resources appropriately, addressing a critical concern in the region and helps to reduce morbidity and mortality.

## 1 Introduction

Malnutrition entails an imbalance between nutrient intake and the essential nutrients required for optimal health and bodily function. It can classified as undernutrition, overnutrition, or micronutrient deficiencies (1). The scope of this review addressed only the burden, spatial distribution and factors associated with underweight and obesity/overweight among lactating women. Underweight is delineated by a body mass index (BMI) of below 18.5 kg/m^2^, while obesity is identified as a BMI exceeding 30 kg/m^2^ (2) and if BMI is between 25 and 29.9 kg/m^2^ it is called overweight. According to prevailing literature in nutrition and women’s health, malnutrition can occur across the life course of women, including adolescence, pregnancy, lactation, and postmenopausal stages, each presenting unique nutritional challenges and health implications (3).

Globally, in 2022, approximately 390 million reproductive women were underweight, while 2.5 billion were deemed overweight and890 million suffered from obesity (4). Underweight in mothers who breastfeed is a serious problem worldwide, especially in developing nations. Research indicates that a significant percentage of lactating mothers are underweight, with prevalence rates in Africa ranging from 1.25 to 30% (5). Malnutrition among lactating mothers poses significant health risks not only to mothers themselves but also to their infants, contributing to intergenerational cycles of undernutrition and poor health outcomes (6). Therefore, this review addressed a critical area of concern within public health and maternal-child nutrition.

Studies have documented regional disparities in malnutrition prevalence. Additionally, research has identified a range of determinants, including socioeconomic factors, cultural practices, and access to healthcare, influencing nutritional outcomes among lactating mothers (7).

However, the extent of evidences about the spatial distribution, burden and factors associated with underweight, obesity and overweight among lactating women at SSA level was unknown. Therefore, this study aimed to extensively review the current evidences on this area and show research gaps which need future investigation.

## 2 Methodology

In this review, peer-reviewed articles, academic dissertations, and reports that described malnutrition among lactating mothers in SSA were searched, reviewed and included. Furthermore, it encompassed observational studies, systematic reviews, and meta-analyses written in English. Lastly, interventional studies were also considered to see programs effectiveness.

A comprehensive exploration spanned various databases, including PubMed, MEDLINE, PsycINFO, HINARI, EMBASE, African Journal of Online (AJOL), Scopus, Web of Science, Google Scholar, and Google. Our search strategy involved a combination of pertinent keywords and Medical Subject Headings (MeSH terms) pertaining to malnutrition, lactating women, and SSA. Identified articles were sifted for determinants according to specified terms. Additionally, primary article references were scrutinized. Statistical analysis was done using Stata version 16 software.

Lastly, the general topic of this research project gyrated around understanding the complex dynamics of malnutrition (underweight, obesity and overweight) among lactating women in SSA who breastfeed their under 2 years child Therefore, studies which conducted to see the spatial distributions, burden and determinants that influence the nutritional status in this population were considered.

## 3 Review results

Double burden of malnutrition (DBM) refers to the simultaneous presence of both undernutrition and overweight or obesity within individuals, households, and populations (8). DBM poses a momentous public health dare in low-and middle-income countries (LMICs) found in Asia (9) and Africa (10).

**Figure 1:**
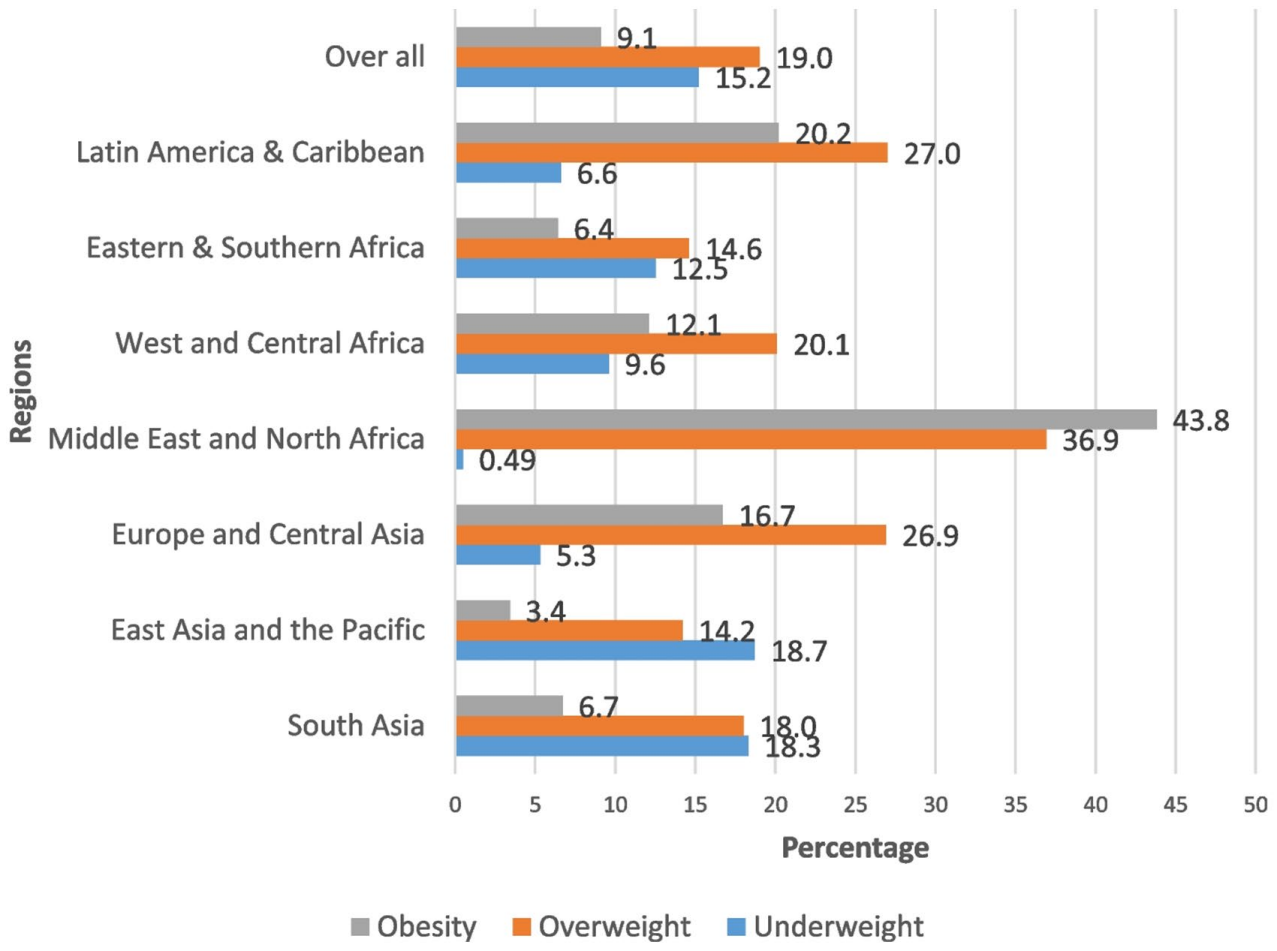
The global burden of malnutrition reported by a study conducted by Alem et al. (11).

As shown in Figure 1, the African region is the most affected (11). In Africa, the DBM among children and women of reproductive age has been extensively studied, with numerous comprehensive and summarized pieces of evidence available (10, 12–21).

However, the extent of evidence available on the malnutrition burden among lactating women was limited. Consequently, the subsequent sections review critically the exact of knowledge on spatial distribution, burden and factors associated with underweight and obesity/overweight among lactating women in SSA.

### 3.1 Spatial distributions of underweight and obesity

Spatial distribution is the arrangement or pattern of objects in physical space and the study of the relationships between them. It is one of the fundamental concepts in geography, urban planning, ecology, health, and other fields (22). By analyzing spatial distribution, researchers can gain insights into processes such as diffusion, migration, urbanization, and resource allocation (23, 24). Population distribution is primarily influenced by a multitude of factors. These include climate, landforms, topography, cultural and political considerations (25).

An analysis using data from the 2016 Ethiopian DHS focused on the geographic distribution of underweight among women of reproductive age identified regions with high burden; Amhara, Tigray, Gambella, and Afar regions (26). Another spatial study conducted among reproductive women in Ethiopia also identified hotspot regions; Tigray, Afar and Amhara (27). Besides, a study conducted in Ethiopia among women also indicated notable hotspot areas in the Eastern and northeastern part of the country (28).

Geospatial research among reproductive-age women in SSA identified significant regions where overweight/obesity and anemia co-occur (29). Several hotspot regions of co-existence were found in Nigeria, South Africa, Mali Cameroon, Mauritania, Tanzania, Liberia and Benin. Conversely, cold spot areas were observed in Burundi, Ethiopia, Guinea, Uganda, Sierra Leone, Madagascar and Rwanda (29). Another study done in Ghana mapped the distribution of undernutrition among non-pregnant women, highlighting clusters in the western Northern region and hotspots of overweight/obesity in several other areas (30).

A nationwide study conducted using data from the Nigerian DHS also examined the spatial distribution of obesity/overweight. It revealed significant variation across ethnic groups and states of residence, with the highest prevalence observed in Cross River State, in southeastern Nigeria, and the lowest in Osun State, situated in southwestern Nigeria (31). All the above studies were conducted among reproductive age women and we did not found any summarized evidence about the spatial distributions of malnutrition among lactating women in SSA. Therefore, a population based geospatial study among lactating women should be conducted using the national DHS data to identify the hotspot countries in the SSA.

### 3.2 Prevalence of underweight among lactating women in SSA

Globally, statistics reveal that approximately 13% of women are underweight. This prevalence is significantly higher in economically disadvantaged nations, with rates ranging from 20 to 25% for underweight (32–34). Pregnant and lactating women are more vulnerable than others (35). The global burden of undernutrition among lactating women is a substantial public health concern, particularly in low-and middle-income countries (LMICs). Recent estimates from the World Health Organization (WHO) indicate that about 22% of lactating women worldwide are affected by undernutrition (36). In SSA countries, this problem presents a grave concern. Consequently, the region grapples with alarmingly high morbidity and mortality rates (37), exacerbated by its susceptibility to both natural and manmade disasters, precipitating socio-economic adversities (5, 38).

**Figure 2:**
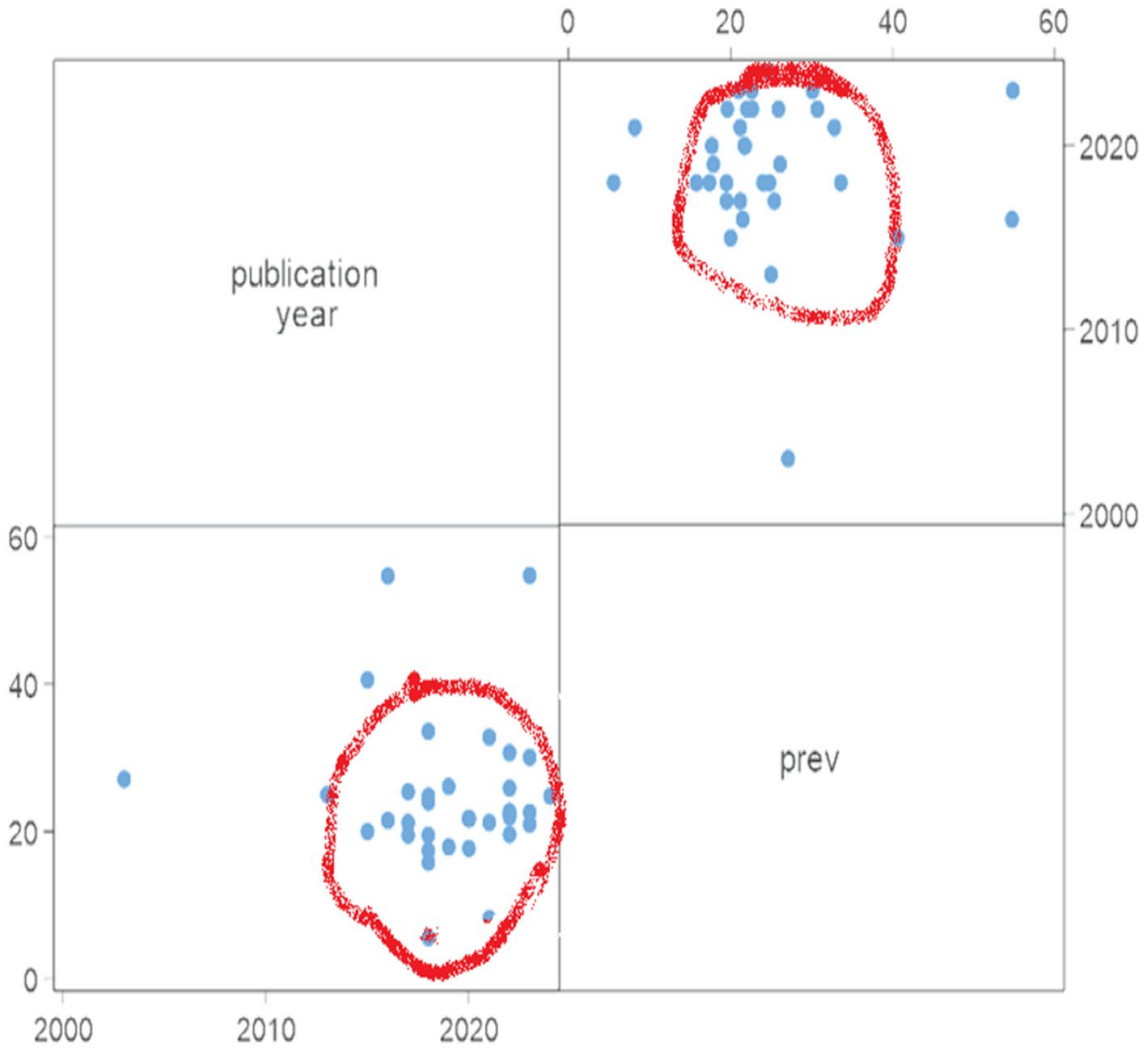
Characteristics of studies conducted in SSA to assess the undernutrition burden among lactating women: reported prevalence and publication year, 2024.

**Figure 3:**
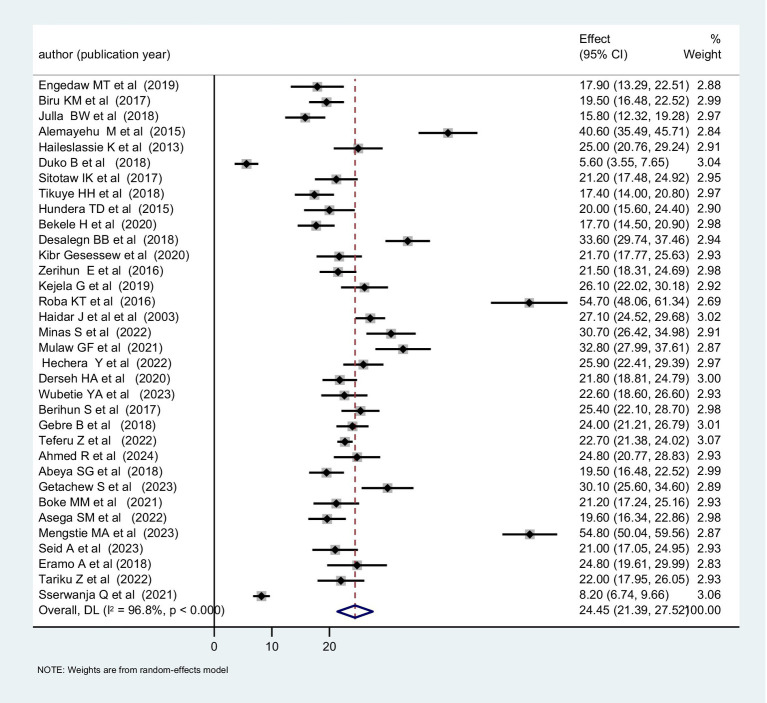
Underweight pooled prevalence in lactating women across SSA, 2024.

The occurrence of undernutrition among lactating women shows considerable variation across different regions and countries, with Sub-Saharan Africa (SSA) shouldering a disproportionate share of the burden. In this region, the prevalence of chronic undernutrition ranges from 10 to 20%, while acute undernutrition ranges from 20 to 25% (39). We analyzed primary studies conducted in Sub-Saharan Africa. Most of these studies were conducted since 2015 and reported a prevalence of undernutrition between 20 and 30% (Figure 2). The combined prevalence was 24.45% (Figure 3).

A study conducted in Uganda using DHS data evaluated the extent of undernutrition among lactating women and revealed that 8.2% of lactating women were underweight (40). Moreover, in Ethiopia, there are over 30 studies have been conducted to gage the extent of undernutrition among lactating women and the prevalence has been found to vary considerably, ranging from 5.6 to 54.8% (41, 42) (Table 1). Another systematic review conducted in Ethiopian also reported a pooled prevalence of underweight of 23.84% (43).

**Table 1:** Studies reported the burden and factors influencing underweight among lactating women in Sub-Saharan Africa, 2024.

Author	Publication year	Country	Design	Sampling	Sample size	prevalence	Measurement	Determinants
Engedaw et al. (64)	2019	Ethiopia	Cross sectional	Systematic random	266	17.9	BMI	Occupation status, feeding frequency and post-natal care
Julla et al. (65)	2018	Ethiopia	Cross sectional	Simple random	422	15.8	BMI	Educational, occupational, marital statuses and family size
Alemayehu et al. (66)	2015	Ethiopia	Cross sectional	Census	355	40.6	BMI	Dietary diversity score
Haileslassie et al. (67)	2013	Ethiopia	Cross sectional	Simple random	400	25	BMI	Length of marriage, ANC frequency, Size of farm land, child age and maize cultivation
Duko et al. (41)	2018	Ethiopia	Cross sectional	Simple random	484	5.6	BMI	Residency, age
Sitotaw et al. (68)	2017	Ethiopia	Cross sectional	Stratified sampling	464	21.2	BMI	Maternal age, extra food during lactation time, occupation and Vitamin A intake
Tikuye et al. (39)	2018	Ethiopia	Cross sectional	Multi stage sampling	478	17.4	BMI	Birth interval, food insecurity, access to nutrition information, workload and educational status.
Biru et al. (69)	2017	Ethiopia	Cross sectional	Simple random	662	19.5	BMI	–
Hundera et al. (70)	2015	Ethiopia	Cross sectional	Simple random	317	20	BMI	Family size and income
Bekele et al. (71)	2020	Ethiopia	Cross sectional	Systematic random	545	17.7	BMI	Dietary diversity, food insecurity, extra meal, place of delivery and income
Kibr et al. (72)	2020	Ethiopia	Cross sectional	Simple random	423	21.7	BMI	Eating motivation, mood concern and meal price
Desalegn et al. (35)	2018	Ethiopia	longitudinal	Multi stage sampling	575	33.6	BMI	–
Zerihun et al. (73)	2016	Ethiopia	Cross sectional	Simple random	638	21.5	BMI	Maternal age, educational status and income
Kejela et al. (74)	2019	Ethiopia	Cross sectional	Systematic random	445	26.1	BMI	Educational status, parity, ANC visit frequency, toilet availability and family size
Roba et al. (75)	2016	Ethiopia	Cross sectional	Simple random	216	54.7	BMI	Parity, region and number of children in the household
Haidar et al. (76)	2003	Ethiopia	Cross sectional	Simple random	1,140	27.1	BMI	–
Sserwanja et al. (40)	2021	Uganda	Cross sectional	Multi stage	1,356	8.2	BMI	No education, not working and region
Minas et al. (77)	2022	Ethiopia	Cross sectional	Multi stage	446	30.7	BMI	Chat chewing, not taking additional weight, and hand washing after toilet use
Mulaw et al. (78)	2021	Ethiopia	Cross sectional	Systematic	366	32.8	BMI	Minimum dietary diversity, and short birth interval
Hechera et al. (79)	2022	Ethiopia	Cross sectional	Simple	607	25.9	BMI	Family size, polygamy, history of abortion and Income
Derseh et al. (80)	2020	Ethiopia	Cross sectional	Multistage	733	21.8	BMI	Dietary diversity, food insecurity, marital status, age at first pregnancy and parity
Wubetie et al. (81)	2023	Ethiopia	Cross sectional	Systematic	420	22.6	BMI	Income, latrine facility, food insecurity, number of meals, dietary diversity and potable water source
Berihun et al. (82)	2017	Ethiopia	Cross sectional	Multistage	668	25.4	BMI	Family size, health education, age of first pregnancy and place of delivery
Gebre et al. (83)	2018	Ethiopia	Cross sectional	Systematic	900	24	MUAC	ANC and feeding support
Teferu et al. (84)	2022	Ethiopia	Cross sectional	Population survey	3,848	22.7	BMI	Age, residence, toilet facilities, and poverty.
Ahmed et al. (85)	2024	Ethiopia	Cross sectional	Multi stage	442	24.8	BMI	Dietary diversity, food insecurity, extra meal and nutrition information
Adugna et al. (86)	2021	Ethiopia	Case control	Consecutive	389	–	BMI	Age at first pregnancy, breastfeeding age and site of delivery,
Abeya et al. (87)	2018	Ethiopia	Cross sectional	Multi stage	662	19.5	BMI	Pregnancy during advice and utilization postnatal service
Getachew et al. (88)	2023	Ethiopia	Cross sectional	Systematic	400	30.1	BMI	Early marriage, extra meal, not using contraceptives, dietary diversity and food insecurity
Boke et al. (89)	2021	Ethiopia	Cross sectional	Simple	410	21.2	BMI	ANC visit, age, income and dietary diversity
Asega et al. (90)	2022	Ethiopia	Cross sectional	Multi-stage	570	19.6	BMI	Family size, age of first pregnancy, health education and maternal education
Mengstie et al. (91)	2023	Ethiopia	Cross sectional	Simple random	420	54.8	BMI	Family size, dietary diversity, birth interval and meal frequency
Seid et al. (92)	2023	Ethiopia	Cross section	systematic	408	21	BMI	Maternal age, marital status, food insecurity and dietary diversity
Eramo (93)	2018	Ethiopia	Cross sectional	Simple random	266	24.8	BMI	ANC follow-up, income, Educational status and extra meals
Tariku et al. (94)	2022	Ethiopia	Cross sectional	Simple random	401	22	BMI	Maternal age, dietary diversity, education status, and family size and extra meal during lactation

However, most of the included studies were conducted in Ethiopia. There wasn’t evidence in the other countries found in SSA and the current combined prevalence could not show the burden in SSA. Therefore, to determine the burden of undernutrition in SSA, a meta-analysis should be conducted using the national DHS data of the member countries since studies included in this study only might not be representative.

### 3.3 Factors influencing underweight in lactating women

Factors contributing to undernutrition among lactating women are multifaceted and include socio-economic disparities, inadequate dietary intake, food insecurity, limited access to maternal healthcare services, and cultural practices (44, 45). Moreover, environmental factors such as climate change and natural disasters exacerbate food insecurity and further compound the risk of undernutrition among vulnerable populations (36).

A mini-review conducted in Ethiopia identified place of delivery, dietary diversity, income, food security, nutritional education programs, and antenatal care as determinants of undernutrition (46). Educational status emerged as a substantial factor for underweight among lactating women in a meta-analysis coxswained in Ethiopia (43).

**Figure 4:**
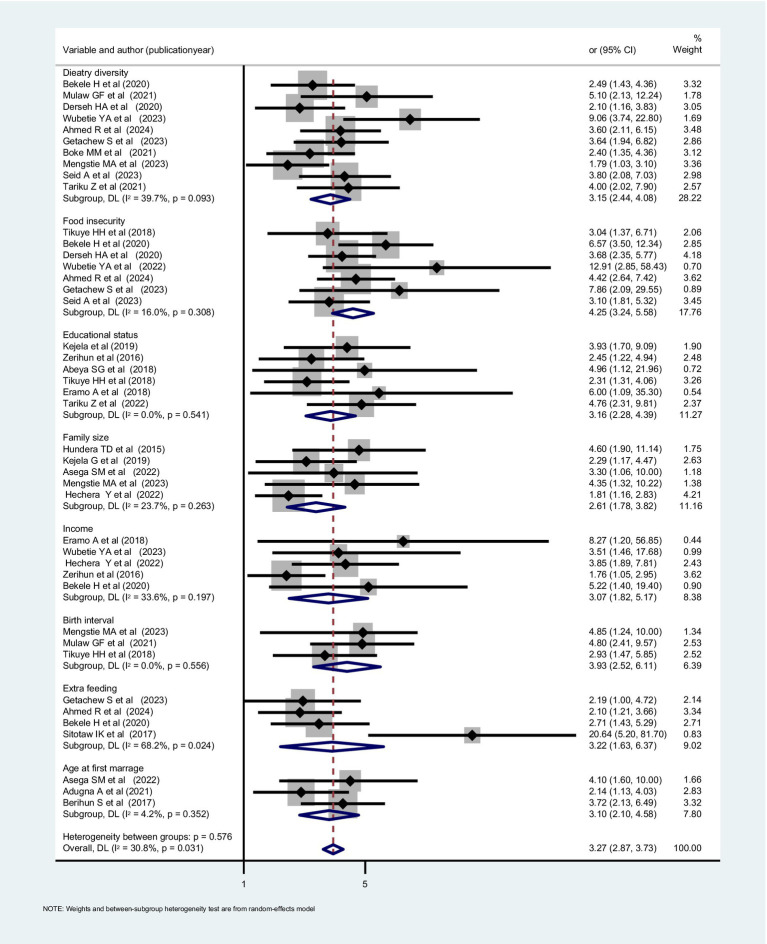
Significant determinants for underweight among lactating women in SSA, 2024.

To reveal determinants that affect nutrition during lactation, different studies conducted in SSA were searched, and many factors that are described in the above paragraphs were scrutinized and included. In summary, the determinants are individual and community-level determinants (Table 1). In our review, the most frequently cited determinants included age at first marriage, dietary diversity score, educational status, family size, food insecurity, income, additional meals, and birth interval (Figure 4).

However, the factors mentioned above are identified from primary local studies, implying they may not fully capture the determinants of undernutrition in SSA. Almost all of the factors are reported from one nation, Ethiopia. Therefore, it is crucial to analyze individual and community-level determinants using comprehensive and representative population-based DHS data of SSA countries. This analysis should employ a multi-level approach, as it is a robust method for understanding the complex and interconnected determinants of undernutrition in SSA (47).

### 3.4 Prevalence and determinants of obesity among lactating women

A study conducted in 32 Sub-Saharan African countries, utilizing DHS data, revealed a pooled obesity prevalence of 6.6% among women. The lowest rate was observed in Madagascar, at 1.1%, while the highest was recorded in Lesotho, at 15.8%. Also, residency and wealth index are reported as determinants for obesity (48).

Another study using DHS data from 24 African countries found increasing obesity rates among urban women of reproductive age. Significant increases were observed across all countries, with notable trends in 17 nations. In Ghana, obesity rates boosted from 1993 to 2014, while Egypt recorded the highest commonness at 39% in 2014. Obesity rates doubled in Burkina Faso, Niger, Benin, Rwanda, Kenya, Ivory Coast, and Uganda and tripled in Malawi, Tanzania, Zambia, and Mali. Conversely, Madagascar and Ethiopia had the lowest obesity prevalence, ranging from 1 to 4% (49).

Furthermore, a separate study conducted in Tanzania documented the obesity prevalence among women, which stood at 9.1% in 2004. This prevalence has shown a consistent and gradual increase over time. Additionally, the study identified age, parity, and socioeconomic status as key determinants of obesity (50).

While studies have explored obesity among women in SSA, the lack of research on the prevalence and determinants of obesity specifically among lactating women in the region is a significant gap. Understanding and addressing obesity among lactating women in SSA, influenced by cultural perceptions, limited access to healthcare resources, and insufficient research focus, could have a profound impact. It has not received the same level of research attention as in other parts of the world (51).

Closing the gap in understanding and addressing obesity among lactating women in SSA requires interdisciplinary efforts involving healthcare professionals, policymakers, researchers, and community stakeholders. This includes promoting culturally sensitive healthcare practices, improving access to healthcare services, prioritizing maternal health and nutrition research, and implementing evidence-based interventions to support healthy weight management during the postpartum period. Furthermore, longitudinal large-scale studies should be conducted to assess the prevalence and determinants of obesity among lactating women in SSA. Addressing these factors can help mitigate the impact of obesity on maternal and child health outcomes in Africa.

### 3.5 Interventional studies conducted in SSA

There were not many interventional studies in sub-Saharan Africa (SSA) that explicitly target obesity, overweight, and underweight among lactating women. Nonetheless, a number of observational studies provide light on the dietary difficulties this demographic faces and offer possible solutions.

According to a Ghanaian study, shorter risks of maternal obesity were linked to longer breastfeeding durations. The odds of being obese were significantly lower for women who breastfed for more than 18 months than for those who did not (52).

The results of observational research indicate that encouraging prolonged breastfeeding may help lower mother obesity, despite the paucity of direct interventional studies. Additionally, resolving the double burden of malnutrition among lactating women in SSA may require addressing socioeconomic variables at the individual and societal levels. There were studies conducted to the rural urban inequality of this problem among lactating women.

There were an additional interventional studies conducted in SSA which displayed on Table 2.

**Table 2:** Summary table for interventional studies conducted among lactating women, 2024.

Country/region	Intervention type	Key outcomes	Reference
Rwanda	Integrated nutrition-specific and nutrition-sensitive program	maternal nutrition knowledge and practices	Habtu et al., 2022 (95)
Uganda	Nutrition Assessment, Counseling, and Support (NACS)	Enhanced maternal nutrition practices	Namukose et al., 2024 (96)
Kenya	Home-based nutritional counseling	exclusive breastfeeding rates	Kimani-Murage et al., 2017 (97)
Ethiopia	Nutrition education and home gardening	Improved dietary diversity	Kuma et al., 2023 (98)

## 4 Discussion

This analytic review paper has aimed to assess the existing knowledge and identify research gaps on the spatial distributions, burden and factors of malnutrition among lactating women in SSA. Even though some studies have been conducted among reproductive-age women at the national level to describe the spatial distribution of malnutrition, no representative research has been conducted focusing on lactating women in SSA. However, investigating the spatial distribution of malnutrition helps to design new interventions and allocate resources, especially for Africa, where the malnutrition burden is high (53). This might be because the region is highly burdened with many communicable and non-communicable diseases. As a result, the concern given to lactating women has become less (54–56).

Although the exact pooled magnitude of underweight among lactating women in SSA remains unknown and most of the included studies were conducted in one country, based on our review, it spans a broad range of 5.6 to 54.8% and the pooled prevalence was 24.45%. This range and pooled prevalence notably exceeds the prevalence reported globally and in studies conducted in developed countries (57). The disparity in prevalence could be attributed to several factors unique to SSA, including disparities in healthcare service quality, lower utilization rates of antenatal and postnatal care, and limited access to nutritional counseling and education compared to developed countries (58, 59).

This review highlights numerous determinants identified by primary studies in SSA, encompassing individual and community-level factors. However, the meta-analysis-based review fails to identify the most pertinent and high-level determinants and their effects. Additionally, the extracted determinants are often sourced from local studies conducted in one country that may not represent broader populations. A population-based multinational study utilizing multilevel analysis of DHS data across SSA countries is warranted to address this gap.

Moreover, our literature review encompasses studies examining the prevalence and factors influencing obesity among women in SSA, revealing a range of 1.1 to 39%. This prevalence is comparable to that observed in high-income countries (60–62), presenting a significant concern and contributing to the DBM in the region. So far, no specific studies have been conducted among lactating women in sub-Saharan Africa. This could be because poverty, food insecurity, and undernutrition provide significant economic obstacles. These significant matters take precedence over obesity treatment, which is frequently perceived as a problem connected to income.

The burden of malnutrition in SSA has been well studied. However, the magnitude was high even compared to other developing countries in Asia, like Bangladesh (wasting of 10%) (63). This could be because a large number of Africans live below the poverty line, there is high food insecurity, and there is conflict in the region.

Despite this was review, it had limitations. Firstly, studies included in this review were mostly from one country which raises generalizability issue.

## 5 Conclusion

This study revealed the absence of evidences on the spatial distribution, burden and factors affecting underweight, obesity and overweight among lactating women in SSA. Moreover, this literature review underscores the critical need for focused attention on malnutrition (underweight and overweight/obesity) among lactating women in SSA. Despite the absence of representative studies in this vulnerable group, our findings reveal a wide prevalence range of underweight and obesity, indicative of significant challenges.

Factors contributing to this include healthcare disparities, limited access to nutritional resources, and the broader burden of disease in the region. Addressing these issues requires a multifaceted approach, including population-based multinational studies and utilization of data from sources like the DHS. Furthermore, the underexplored prevalence of obesity among lactating women underscores the urgent need for further research. This research is crucial to thoroughly understanding the extent of this issue and effectively addressing the problem in this vulnerable population. Identifying hotspot areas of malnutrition specifically among lactating women within sub-Saharan Africa through spatial distribution analysis is essential for allocating resources appropriately, addressing a critical concern in the region and helps to reduce morbidity and mortality. Therefore, to fill this gap studies using representative datasets like DHS data at SSA level is essential and future researchers should also give focus to lactating women nutrition since they are more vulnerable.
